# Morphological and moisture availability controls of the leaf area-to-sapwood area ratio: analysis of measurements on Australian trees

**DOI:** 10.1002/ece3.1344

**Published:** 2015-02-25

**Authors:** Henrique Furstenau Togashi, Iain Colin Prentice, Bradley John Evans, David Ian Forrester, Paul Drake, Paul Feikema, Kim Brooksbank, Derek Eamus, Daniel Taylor

**Affiliations:** 1 Department of Biological Sciences, Macquarie UniversitySydney, New South Wales, Australia; 2 AXA Chair of Biosphere and Climate Impacts, Grand Challenges in Ecosystems and the Environment and Grantham Institute – Climate and Environment, Department of Life Sciences, Imperial CollegeLondon, UK; 3 Department of Forest and Ecosystem Science, The University of MelbourneMelbourne, Victoria, Australia; 4 Chair of Silviculture, Faculty of Environment and Natural Resources, Freiburg UniversityFreiburg, Germany; 5 Natural Resources Branch, Department of Parks and Wild LifeBentley, Western Australia, Australia; 6 School of Plant Biology, University of Western AustraliaCrawley, Western Australia, Australia; 7 Agricultural Resource Risk Management, Department of Agriculture and FoodAlbany, Western Australia, Australia; 8 School of Environment, University of Technology SydneySydney, New South Wales, Australia; 9 National Centre for Groundwater Research and TrainingSydney, New South Wales, Australia

**Keywords:** Climatic moisture, leaf area, pipe model, plant hydraulics, sapwood area, tree morphology

## Abstract

1. The leaf area-to-sapwood area ratio (*LA:SA*) is a key plant trait that links photosynthesis to transpiration. The pipe model theory states that the sapwood cross-sectional area of a stem or branch at any point should scale isometrically with the area of leaves distal to that point. Optimization theory further suggests that *LA:SA* should decrease toward drier climates. Although acclimation of *LA:SA* to climate has been reported within species, much less is known about the scaling of this trait with climate among species.
2. We compiled *LA:SA* measurements from 184 species of Australian evergreen angiosperm trees. The pipe model was broadly confirmed, based on measurements on branches and trunks of trees from one to 27 years old. Despite considerable scatter in *LA:SA* among species, quantile regression showed strong (0.2 < *R*1 < 0.65) positive relationships between two climatic moisture indices and the lowermost (5%) and uppermost (5–15%) quantiles of log *LA:SA*, suggesting that moisture availability constrains the envelope of minimum and maximum values of *LA:SA* typical for any given climate.
3. Interspecific differences in plant hydraulic conductivity are probably responsible for the large scatter of values in the mid-quantile range and may be an important determinant of tree morphology.

## Introduction

Trade-offs between plant functional traits are central to optimality theories developed to account for observed covariation among traits (Wright et al. 2004; Warton et al. 2006). Investigations of trait variation under different environmental conditions can yield insight into how plant strategies adapt to specific habitats and provide information for modeling plant growth in response to environmental change (Mencuccini and Bonosi, 2001). Here, we focus on the relationships between two critically important traits for the function of trees: leaf area (*LA*), which determines tree water use, light interception, and thus photosynthesis, and sapwood area (*SA*), which determines hydraulic capacity and thus the tree's ability to supply water to the leaves.

According to the “pipe model” (Shinozaki et al. 1964), the leaf area of a stem or branch should be proportional to (scale isometrically with) the sapwood cross-sectional area that sustains it (Tyree and Ewers 1991). As the construction and maintenance of sapwood entail substantial costs, optimization theory further predicts that the ratio *LA:SA* should adjust to environmental conditions in such a way as to maximize photosynthetic revenue relative to these costs. In dry environments, high vapor pressure deficits (*vpd*) generally imply that larger transpiration rates have to be maintained in order to achieve a given rate of photosynthesis. As a result, the optimal ratio of stomatal conductance to photosynthetic capacity (minimizing the combined costs of maintaining the capacity for both transpiration and carboxylation) is lower in drier environments (Wright et al. 2004; Prentice et al. 2014). However, usually a larger sapwood area per unit area of transpiring leaf has to be maintained if photosynthetic rates are to equal those achieved in wetter environments (Westoby et al. 2012). Thus, we expected to find a trend toward systematically lower *LA:SA* ratios with increasing aridity.

We combined new field measurements of *LA:SA* ratios with published values and values from unpublished studies at sites across a range of Australian environments, from wet to semi-arid and cool-temperate to tropical climates. We used these data – which come from trees of different heights, and include measurements on whole trees as well as branches – to test the general scaling relationships between *LA* and *SA*. We then analyzed relationships between *LA:SA* and several indices of climatic moisture in order to test the predicted relationships between *LA:SA* and aridity. We confined attention to evergreen angiosperms, the dominant tree functional type in Australia. Gymnosperm wood has very different hydraulic properties, while tropical deciduous trees would be expected to respond only to wet-season conditions and therefore to show different patterns of variation with climate.

## Methods

### Measurements and data synthesis

We measured *LA* and *SA* on four branches per tree of ten evergreen angiosperm species at Robson Creek, Danbulla National Park, Queensland (17°07′S, 145°37′E). Leaf area was measured using a desktop scanner for all leaves on a sampled branch. After bark removal, the branch cross-sectional area was measured with a digital caliper at two points near the cut (methodology that includes the nonconductive part of the sapwood). The pith cross-sectional area was measured and subtracted from the branch cross-sectional area.

**Figure 1:**
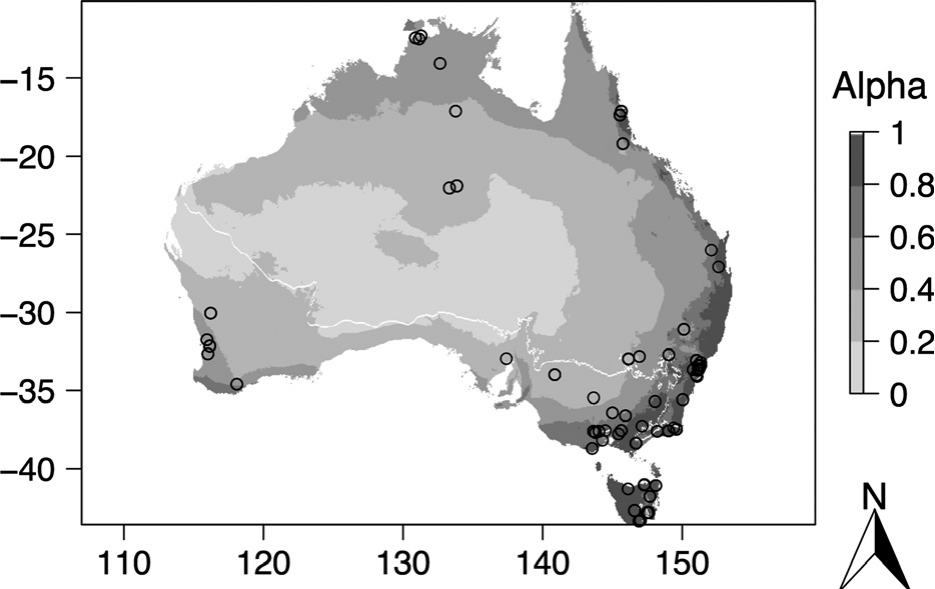
Geographic distribution of measured leaf area-to-sapwood area (*LA:SA*) ratios. The mapped climate variable is the Cramer–Prentice *α* index of moisture availability (average between 1970 and 2000) calculated from AWAP data (http://www.eoc.csiro.au/awap/). The white line shows the limit between May to September north dry season and October to April south dry season.

Data for a further 170 species were contributed from 25 sites, based on seven published and two unpublished studies. We also compiled 258 published measurements on 151 species from 43 sites (Supplementary Information Data S1 and S2). The data comprised measurements from 183 species, at the locations shown in Fig.1. The full dataset was composed of 252 measurements on branches and 175 on whole trees. For compiled and contributed data, measurements on branches were made as described above, although with a variable number of branches sampled per tree. In some studies where the branch diameter was too small (<10 mm), the pith was considered part of the sapwood. For whole trees, cross-sectional sapwood area was measured at 1.3 m above the ground from bored cores or harvesting the tree. Leaf area in whole trees was in most cases measured from harvested trees. Data for individual height in 38 species (49 observations) were also gathered. A further subset was considered for 35 species (101 observations) for which xylem-specific hydraulic conductivity (*K*_s_) was available simultaneously with LA:SA and climatic moisture.

### Climate data

Mean *vpd* was estimated at each sampling site from the Bureau of Meteorology (BoM) 0.01-degree gridded climatology using seven models (Running and Coughlan 1988; Allen et al. 1998; Wang et al. 2004), all of which yielded similar results. For illustration, we present results obtained using the equation of Allen et al. (1998), also adopted by the Australian Bureau of Meteorology (BoM), http://www.bom.gov.au:

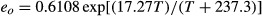


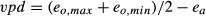


where *T* is daily temperature (°C), *e*_*o, max*_ is the saturated vapor pressure (kPa) calculated at the daily maximum temperature, *e*_*o, min*_ is the saturated vapor pressure calculated at the daily minimum temperature, and *e*_*a*_ is the mean daily actual vapor pressure, calculated from the BoM product as the average of measurements at 0900 and 1500 h.

Two additional climatic indicators of moisture availability were calculated, using Australia Water Availability Project (AWAP) gridded data (http://www.eoc.csiro.au/awap/): the moisture index (*MI*) (the ratio between precipitation and potential evaporation) and the Cramer–Prentice *α* index (Prentice et al. 2011) (calculated here as the ratio between actual and potential evaporation in the AWAP data). Both *MI* and *α* are dimensionless numbers and are conceptually related by the Budyko curve, whereby *α* is a saturating function of *MI*.

All moisture variables (including *vpd*) were calculated for months, years, decades, and dry seasons (the hydraulic architecture of evergreen species may sometimes present a “bottleneck” effect as response to dry season). “Dry season” was defined following the BoM convention as May to September in the north and October to April in the south. The Australian averaged rainfall gridded values for the northern dry season months were subtracted from the averaged southern dry season (1970–2000). Negative values were considered “May to September north dry season” and positive values “October to April south dry season” (Fig.1).

### Data analysis

We tested the pipe model by regressing *LA* against *SA* values for branches and trunks, between species and across environments, using standardized major axis (SMA) regression with the *smatr* package (Warton et al. 2006) in the R language (R Core Team 2012). SMA is a least-squares method which fits the first major axis to standardized data and then retransforms the data to their original scales. The method is useful for studies of the relationships between different dimensions and traits of organisms where natural variability is expected to be present in both variables, as it handles them in a symmetrical way (Warton et al. 2006).

Ordinary linear regression was used to fit bivariate relationships between log *LA:SA* and climatic moisture (*α*, *MI* and *vpd*). Multiple regression was used for analyses exploring the additional predictive power of plant height and for LA:SA, *K*_s_, and climatic moisture comparisons. The *quantreg* package (Koenker 2004) in R was used to perform quantile regressions between log *LA:SA* and climate variables. Quantile regression (Koenker and Bassett 1978) provides a more complete understanding of relationships between variables than can be obtained with simple regression, allowing different slopes to apply in different quantiles of the dependent variable. Is it also particularly useful when analyzing datasets with unequal variation (Cade and Noon 2003). The coefficient of determination associated with quantile regression is based on absolute rather than squared deviations. For any given quantile *τ*, this “pseudo-*R*^2^” is *R*1 = 1 − *F*(*τ*)/*R*(*τ*), where *F*(*τ*) is the weighted sum of absolute deviations from the regression model and *R*(*τ*) is the weighted sum of absolute deviations from the corresponding model with zero slope (Koenker and Machado 1999).

## Results

### Branch to trunk allometry

**Figure 2:**
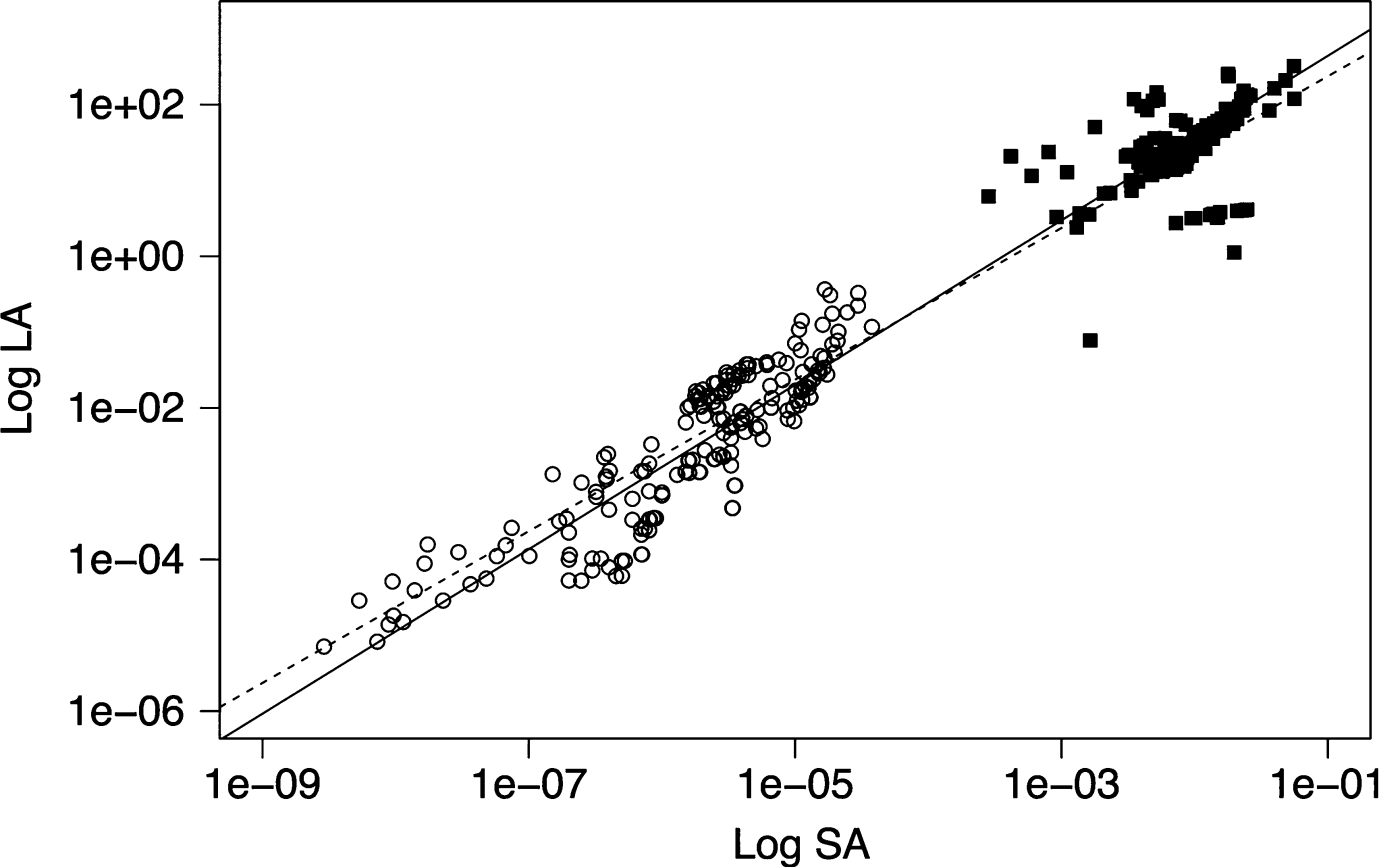
Standardized major axis (SMA) regression between log-transformed leaf area (log *LA*) and sapwood area (log *SA*) for 184 tree species across Australia. Units are in m^2^. Open circles and filled squares are observations in branches and whole trees, respectively. The solid line represents the SMA linear regression, and the dashed line shows the line obtained when the slope was constrained to 1. The allometric equations are given in the text.

Leaf area and sapwood area are both necessarily larger for whole trees than for individual branches. The two variables were strongly and positively correlated across scales (SMA regression: log *LA* = 1.08 log *SA* + 3.72, *R*^2^ = 0.94, *P* < 0.05) (Fig.2) with a slope slightly, but significantly (*P* < 0.01), larger than unity. This finding indicates that while the measurements follow a relationship close to the pipe model, there is a systematic tendency for the ratio *LA:SA* to be higher for whole trees.

A generic (cross-species) estimate of *LA:SA* was obtained by constraining the log–log slope to 1, yielding an intercept of 3.3 (*P* < 0.05) corresponding to a ratio of 2363 ± 818 m^2^ m^−2^. SMA regression also indicated equal slopes for log *LA versus* log *SA* in trunks and in branches (*P* > 0.05 for the null hypothesis of equality) but different intercepts (*P* < 0.05). Constraining the slopes to 1 yielded an intercept of 3.28 (*P* < 0.05), corresponding to a generic *LA:SA* of 1933 ± 578, for branches, and 3.49 (*P* < 0.05), corresponding to a generic *LA:SA* of 3093 ± 980, for whole trees.

### Leaf area-to-sapwood area ratio and tree height

**Figure 3:**
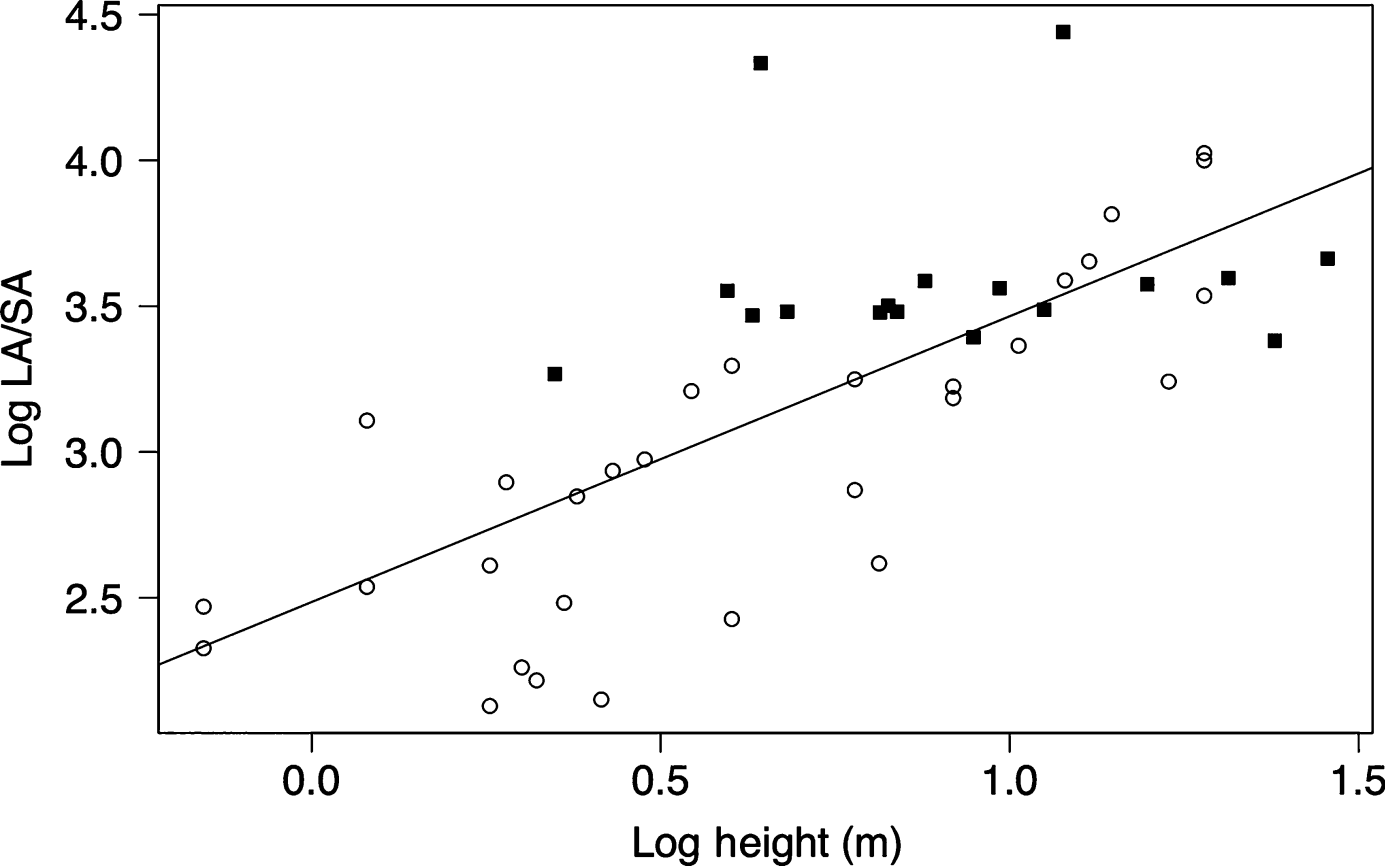
Relationship between log-transformed leaf area-to-sapwood area ratio (log *LA*/*SA*) and log tree height in 39 angiosperm tree species. Open circles and filled squares are observations in branches and whole trees, respectively.

There was a significant positive correlation between log *LA:SA* and log tree height (*R*^2^ = 0.52, *P* < 0.05) (Fig.3). The correlation was stronger for branches only (*R*^2^ = 0.64, *P* < 0.05). There was no significant relationship for whole trees (slope 0.001, *P* > 0.05) (lines not shown). The overall increase in *LA:SA* with increasing tree height seems to be ontogenetic, and not an artifact of correlation between height and climatic moisture, as there was no significant bivariate relationship between tree height and climatic moisture (*MI*, *α* or *vpd*). A multiple linear regression of height and each of the climatic moisture variables as predictors for *LA:SA* (*R*^2^ = 0.24) returned a significant slope for height (*P* < 0.05) but a nonsignificant slope for climatic moisture (*P* > 0.05) (graphs not shown).

### Relationship of LA:SA to climatic moisture

**Figure 4:**
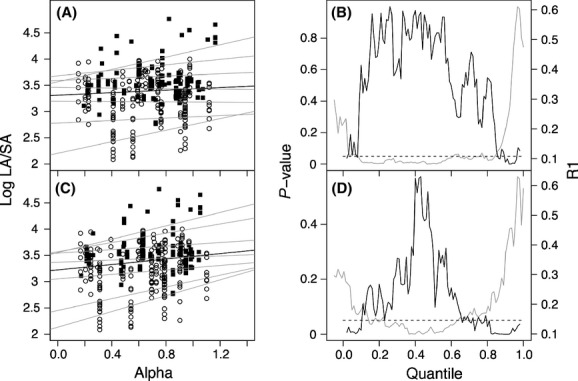
Filled points and open circles are *LA*/*SA* observations in whole trees and branches, respectively. The left panels show quantile linear regressions lines in gray (*τ *= 0.05, 0.1, 0.25, 0.5, 0.75, 0.9, 0.95) and ordinary least-squares linear regression line in black, for log-transformed leaf area-to-sapwood area ratio (log *LA*/*SA*) as a function of annual (A) and decadal (C) values of *α*. The right panels show the corresponding *P*-values (black) and pseudo *R*-squared (*R*1) values (gray) as a function of *τ*.

The overall linear regression relationships of log *LA:SA* to annual, decadal, dry-season, and mean monthly *α* (Fig.4) were not statistically significant. Linear regressions between log *LA:SA* and *MI* and mean *vpd* were also not significant. However, the upper and lower bounds of log *LA:SA* tend to increase with increasing *α* even though the cloud of points does not show such a relationship (Fig.4, left panels). Quantile regressions confirmed this, with *α* accounting for about 0.2 of the bottom 7% (*P* < 0.05) and from 0.3 to 0.55 of the top 15% (*P* < 0.05) of the variation in *LA:SA* (Fig.4, right panels). Similarly, *MI* accounted for 0.2 to 0.35 of the top 10% (*P* < 0.05) of the observed variation in *LA:SA* (data not shown). In contrast, dry season and monthly *α* and *MI* could not explain any quantiles of the relationship (data not shown). There were also no significant relationships between *LA:SA* and *vpd* (*R*1 < 0.2, *P* > 0.05) for any *τ* (data not shown).

These relationships were valid whether *LA:SA* was measured in branches (for the lower end) or in whole trees (for the upper end). No value of log *LA:SA* in whole trees was smaller than 2.7 (502 < *LA:SA* < 58 200), while values of log *LA:SA* in branches were generally restricted to between 2 and 4 (122 < *LA*/*SA* < 10 000). Part of the spread in values is due to the slight departure from isometry, such that whole trees have larger values than branches. Significant correlations between log *LA:SA* and *α* in this dataset are restricted to the upper and the lower quantiles where there are either only whole-tree or only branch measurements, respectively. Nevertheless, when separate regressions were performed for whole trees and branches, the former yielded similar significant relationships for the upper quantiles and the latter yielded similar significant relationships for the lower quantiles.

### Relationship of LA:SA to K_s_ and climatic moisture

**Figure 5:**
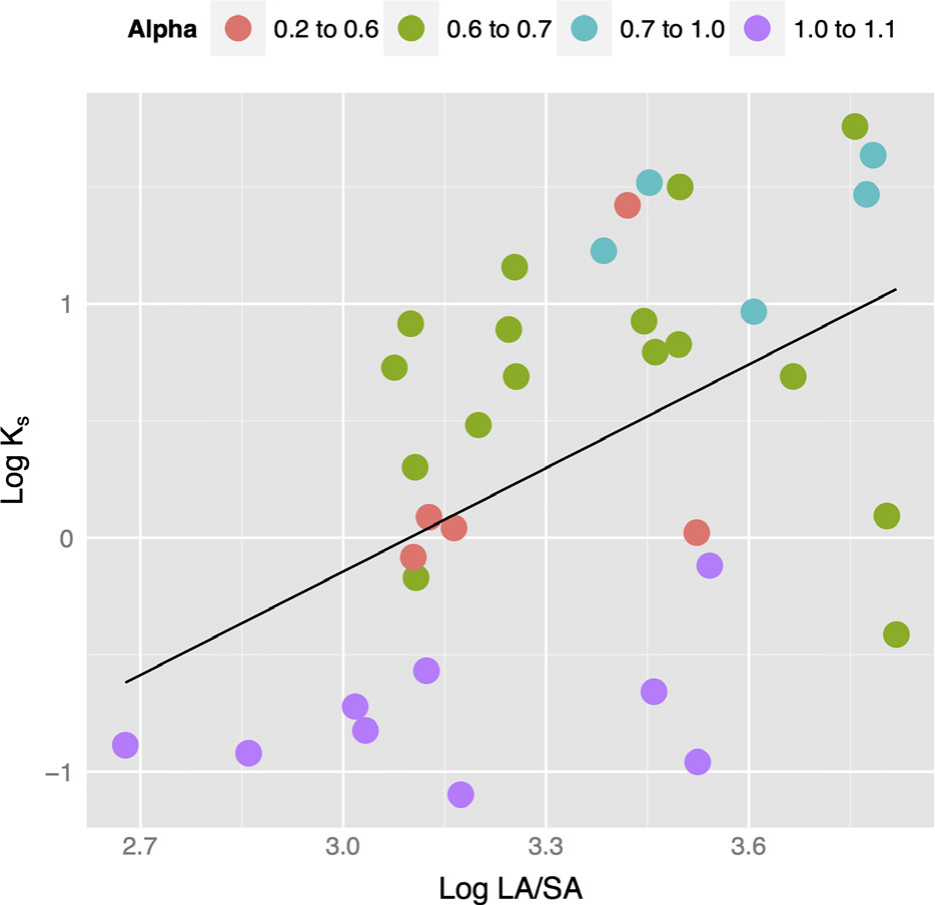
Linear regression of xylem-specific hydraulic conductivity (*K*_s_) and *LA/SA*. Values of the Cramer–Prentice Alpha (*α*) index (*R*^2^ = 0.34, *P* < 0.05) are superimposed.

*K*_s_ data were available only in the higher range of log *LA:SA* values (between 2.6 and 4). Within this range, xylem-specific hydraulic conductivity showed some predictive ability for *LA:SA*. The bivariate linear regression was significant (*P* < 0.05), and *R*^2^ was 0.24 (Fig.5). The positive regression slope indicates an increase in *LA:SA* with increasing *K*_s_. Including the index *α* in a multiple regression increased the correlation to *R*^2^ = 0.34 (*P* < 0.05). A model including an interaction term (*K*_s_:*α*) also returned significant results for alpha (*P* < 0.05). Species in wetter environments present higher LA:SA at a given *K*_s_ than species in drier environments.

## Discussion

### LA to SA allometry in branches and whole trees

The close, near-isometric relationship (slope 1.08 ± 0.02) between leaf area and sapwood area shown in Fig.2 is broadly consistent with the pipe model. The similar slopes and different intercepts between *LA* and *SA,* however, indicate a tendency for overall higher *LA:SA* ratios at the whole-tree scale. This conclusion is supported by our generic regression-based estimates of the *LA:SA* ratio, which are about 50% higher for whole trees than for branches. The difference could perhaps be an artifact of the assumption, made in many studies, that branches are composed entirely of sapwood (i.e., the pith is not accounted for). Alternatively, it could be explained by the variations in the relationship of *LA:SA* with tree height and climatic moisture in plant hydraulic traits.

### Relationship of LA:SA to tree height

If hydraulic properties of wood did not change with tree size, the hydraulic conductivity of the stem would be in inverse proportion to its height. This could have two possible consequences. One would be that tall trees would have much reduced rates of transpiration per unit of leaf area. The other would be a requirement for large reductions in *LA:SA* with height, to compensate for the increased resistance to water transport – in direct contradiction to the pipe model. Neither of these effects is observed, because of the phenomenon of basipetal tapering of conducting tissues (Tyree and Ewers 1991; Olson and Rosell 2013). Olson et al. (2014) have shown that this is universal in the angiosperms. Taller plants are associated with larger vessel diameters but lower vessel densities (Olson et al. 2014). Increasing vessel diameter is a highly effective means of maintaining hydraulic conductivity, because of the fourth-power dependence of conductivity on vessel diameter, whereas hydraulic path length scales in the linear dimension according to Poiseuille's law (Tyree and Ewers 1991; Sellin and Kupper 2006). Olson and Rosell (2013) and Olson et al. (2014) demonstrated that vessel diameter is strongly predicted by stem diameter and that species in drier environments tend to have narrower vessels simply because they are shorter. Olson et al. (2014) further demonstrated that direct climate controls on vessel diameter are very weak, compared with the pure effect of size.

Low irradiances may favor height growth over an increase in sapwood area in plants not adapted to low light, where competition for light is intense (Calvo-Alvarado et al. 2008). For many tree species, height growth is an important process to outcompete neighbors for sunlight. Increased water transport through wider vessels with increasing height would provide a selective advantage when water is not limiting. There will always be some individual trees with small vessel density and some with large vessel density, and when conditions are favorable, trees may just increase the proportion of large-diameter vessels. Even if vessel density related to height and not to climate, height should correlate with moisture availability; thus, the apparent trade-off between vessel density and climatic moisture is important for modeling purposes.

Tree height was positively correlated with *LA:SA* (Fig.3) in contrast to several other studies in angiosperms (Schäfer et al. 2000; Sellin and Kupper 2006) and conifers (Magnani et al. 2000; McDowell et al. 2002; Delzon et al. 2004) where the correlation was found to be negative. All those studies were conducted in temperate climates. Other studies have shown *LA:SA* increasing with size for angiosperms in warm moist environments (Gerrish 1990; Vertessy et al. 1995; Mokany et al. 2003; Calvo-Alvarado et al. 2008), for conifers generally (McDowell et al. 2002), and for angiosperms in seasonal climates (Phillips et al., 2003). For exceptions in angiosperms, see Calvo-Alvarado et al. (2008) where one species of five showed a decrease in *LA:SA* with height in a wet environment, and for gymnosperms, see Hubbard et al. (1999) and Long and Smith (1988) where *LA:SA* increased with height in a dry environment. Considering the results in the present study and the generally similar results in the literature, it may be that the relation between tree height and *LA:SA* will be directly or indirectly influenced by environmental conditions: Warm moist environments should thus show positive correlations, and cold enviroments should show negative correlations. A more systematic global survey would be needed to test this.

### Relationships of LA:SA to climatic moisture and the role of xylem hydraulic conductivity

There is evidence for acclimation of *LA:SA* across sites within *Pinus* species (Mencuccini and Grace 1995; Delucia et al. 2000; Schäfer et al. 2000; Warton et al. 2006). In contrast, relatively little has been published about how *LA:SA* among angiosperm species is affected by climatic characteristics of contrasting environments. Yet this information is required when modeling carbon and plant trait trade-offs in ecosystems.

Every growing season provides an opportunity for trees to modify *LA:SA*. The dimensions of xylem elements are one of the most important traits to determine a conservative or profligate strategy of water use, and this underpins the potential carbon gain attainable over the season (Magnani et al. 2000). The narrow tracheids of gymnosperms imply a low hydraulic conductivity, but this is accompanied by a reduced risk of embolism and thus the capacity to adapt to dry environments and also to environments subject to freezing (Tyree and Ewers 1991; Delucia et al. 2000). The low conductivity of gymnosperm wood is compensated for by larger sapwood area and generally smaller *LA:SA* in order to maintain rates of water transport. Variation in diameter of xylem-conducting elements between angiosperm species is much wider than between gymnosperm species (Sperry et al., 2006), and this fact may well have contributed to the large variation in *LA:SA* that we observed. Contrasts between low *LA:SA*, usually accompanied by narrow crowns in gymnosperm trees and high *LA:SA*, accompanied by more widely spreading crowns in many angiosperm trees, is an important aspect of the morphological difference between these phyla which may ultimately be controlled by differences in hydraulic capacity.

We could not confirm any universal relationship between *LA:SA* and moisture availability (Fig.4). Non-*Pinus*-conifers showed a similar lack of relationship across environments at a global scale (Delucia et al. 2000). Nevertheless, quantile regression revealed a systematic increase in both the upper and lower bounds of *LA:SA* with increasing climatic moisture, as indexed by *α* or *MI*. Moreover, because of the slight departure from isometry in the relationship between *LA* and *SA*, the upper bound is formed by whole trees and the lower bound by branches. But the importance of these bounds is dwarfed by the large variation in *LA:SA* found within both tree and branch measurement sets. This variation may be related to plant hydraulic conductivity, which also shows large variations among species and which has frequently been mentioned as a key trait coordinated with leaf area and sapwood area to achieve convergence in water use (Mencuccini and Grace 1995; Zeppel and Eamus 2008; Choat et al. 2012; Gleason et al. 2012). Higher ranges of *LA:SA* in Australian angiosperm trees could indeed be predicted by xylem-specific hydraulic conductivity, and this relationship was stronger when considering climatic moisture. However, the dataset available for this study could not confirm a more general correlation in all ranges of *LA:SA* (Fig.5). Improved understanding of the spectrum of plant hydraulic function would be promoted by studies specifically orienteded to coordinated measurements of *LA:SA* and hydraulic traits across species and environments.

## Supporting Information

**Data S1.** Togashi et al. Moisture availability constraints on LA:SA dataset.

**Data S2.** List of published literature for data compilation.
