# Supplementary material for: Morphological and moisture availability controls of the leaf area-to-sapwood area ratio: analysis of measurements on Australian trees
Source: Ecol Evol. 2015 Feb 25;5(6):1263–70. doi: 10.1002/ece3.1344 (PMC4377269; doi:10.1002/ece3.1344)
Supplement: Supplementary file 2 [file ece30005-1263-sd2.docx]

**List of published literature for data compilation**

BARRETT, D. J., HATTON, T. J., ASH, J. E. & BALL, M. C. 1996. Transpiration by Trees From Contrasting Forest Types. *Australian Journal of Botany,* 44**,** 249-263.

BENYON, R. G., MARCAR, N. E., CRAWFORD, D. F. & NICHOLSON, A. T. 1999. Growth and water use of Eucalyptus camaldulensis and E. occidentalis on a saline discharge site near Wellington, NSW, Australia. *Agricultural Water Management,* 39**,** 229-244.

BLEBY, T. M., COLQUHOUN, I. J. & ADAMS, M. A. 2009. Architectural plasticity in young Eucalyptus marginata on restored bauxite mines and adjacent natural forest in south-western Australia. *Tree Physiology,* 29**,** 1033-1045.

BRODRIBB, T. J. & FEILD, T. S. 2000. Stem hydraulic supply is linked to leaf photosynthetic capacity: evidence from New Caledonian and Tasmanian rainforests. *Plant Cell & Environment,* 23**,** 1381-1388.

BROOKSBANK, K., VENEKLAAS, E. J., WHITE, D. A. & CARTER, J. L. 2011. Water availability determines hydrological impact of tree belts in dryland cropping systems. *Agricultural Water Management,* 100**,** 76-83.

CANHAM, C. A., FROEND, R. H. & STOCK, W. D. 2009. Water stress vulnerability of four Banksia species in contrasting ecohydrological habitats on the Gnangara Mound, Western Australia. *Plant Cell & Environment,* 32**,** 64-72.

CARTER, J. L. & WHITE, D. A. 2009. Plasticity in the Huber value contributes to homeostasis in leaf water relations of a mallee Eucalypt with variation to groundwater depth. *Tree Physiology,* 29**,** 1407-18.

CERNUSAK, L. A., HUTLEY, L. B., BERINGER, J. & TAPPER, N. J. 2006. Stem and leaf gas exchange and their responses to fire in a north Australian tropical savanna. *Plant Cell & Environment,* 29**,** 632-646.

CHOAT, B., BALL, M. C., LULY, J. G. & HOLTUM, J. A. M. 2005. Hydraulic architecture of deciduous and evergreen dry rainforest tree species from north-eastern Australia. *Trees - Structure and Function,* 19**,** 305-311.

DRAKE, P. L. & FRANKS, P. J. 2003. Water resource partitioning, stem xylem hydraulic properties, and plant water use strategies in a seasonally dry riparian tropical rainforest. *Oecologia,* 137**,** 321-329.

DRAKE, P. L., FROEND, R. H. & FRANKS, P. J. 2011. Linking hydraulic conductivity and photosynthesis to water-source partitioning in trees versus seedlings. *Tree Physiology,* 31**,** 763-773.

EAMUS, D., O'GRADY, A. P. & HUTLEY, L. 2000. Dry season conditions determine wet season water use in the wet–tropical savannas of northern Australia. *Tree Physiology,* 20**,** 1219-1226.

ELDRIDGE, S., THORBURN, P.J., MCEWAN, K.L. AND HATTON T.J. 1993. Health and structure of Eucalyptus communities on Chowilla and Monoman Islands of the River Murray floodplain, South Australia / by Steven R. Eldridge ... [et al.]. *Divisional report (CSIRO. Division of Water Resources) ; 93/3.*

FEIKEMA, P. M., MORRIS, J. D., BEVERLY, C. R., COLLOPY, J. J., BAKER, T. G. & LANE, P. N. J. 2010. Validation of plantation transpiration in south-eastern Australia estimated using the 3PG+ forest growth model. *Forest Ecology and Management,* 260**,** 663-678.

FORRESTER, D. I., COLLOPY, J. J., BEADLE, C. L. & BAKER, T. G. 2012. Interactive effects of simultaneously applied thinning, pruning and fertiliser application treatments on growth, biomass production and crown architecture in a young Eucalyptus nitens plantation. *Forest Ecology and Management,* 267**,** 104-116.

FORRESTER, D. I., COLLOPY, J. J. & MORRIS, J. D. 2010a. Transpiration along an age series of Eucalyptus globulus plantations in southeastern Australia. *Forest Ecology and Management,* 259**,** 1754-1760.

FORRESTER, D. I., THEIVEYANATHAN, S., COLLOPY, J. J. & MARCAR, N. E. 2010b. Enhanced water use efficiency in a mixed Eucalyptus globulus and Acacia mearnsii plantation. *Forest Ecology and Management,* 259**,** 1761-1770.

KALMA, S. J., THORBURN, P. J. & DUNN, G. M. 1998. A comparison of heat pulse and deuterium tracing techniques for estimating sap flow in Eucalyptus grandis trees. *Tree Physiology,* 18**,** 697-705.

MCCLENAHAN, K., MACINNIS-NG, C. & EAMUS, D. 2004. Hydraulic architecture and water relations of several species at diverse sites around Sydney. *Australian Journal of Botany,* 52**,** 509-518.

MEDHURST, J. L., BATTAGLIA, M. & BEADLE, C. L. 2002. Measured and predicted changes in tree and stand water use following high-intensity thinning of an 8-year-old Eucalyptus nitens plantation. *Tree Physiology,* 22**,** 775-784.

MEDHURST, J. L., BATTAGLIA, M., CHERRY, M. L., HUNT, M. A., WHITE, D. A. & BEADLE, C. L. 1999. Allometric relationships for Eucalyptus nitens (Deane and Maiden) Maiden plantations. *Trees - Structure and Function,* 14**,** 91-101.

MOKANY, K., MCMURTRIE, R. E., ATWELL, B. J. & KEITH, H. 2003. Interaction between sapwood and foliage area in alpine ash (Eucalyptus delegatensis) trees of different heights. *Tree Physiology,* 23**,** 949-958.

MORRIS, J. D. & COLLOPY, J. J. 1999. Water use and salt accumulation by Eucalyptus camaldulensis and Casuarina cunninghamiana on a site with shallow saline groundwater. *Agricultural Water Management,* 39**,** 205-227.

O’GRADY, A., COOK, P., EAMUS, D., DUGUID, A., WISCHUSEN, J., FASS, T. & WORLDEGE, D. 2009. Convergence of tree water use within an arid-zone woodland. *Oecologia,* 160**,** 643-655.

O’GRADY, A. P., WORLEDGE, D. & BATTAGLIA, M. 2006. Above- and below-ground relationships, with particular reference to fine roots, in a young Eucalyptus globulus (Labill.) stand in southern Tasmania. *Trees,* 20**,** 531-538.

PICKUP, M., WESTOBY, M. & BASDEN, A. 2005. Dry mass costs of deploying leaf area in relation to leaf size. *Functional Ecology,* 19**,** 88-97.

PINKARD, E. A. & NEILSEN, W. A. 2003. Crown and stand characteristics of Eucalyptus nitens in response to initial spacing: implications for thinning. *Forest Ecology and Management,* 172**,** 215-227.

PRIOR, L. D. & EAMUS, D. 2000. Seasonal changes in hydraulic conductance, xylem embolism and leaf area in Eucalyptus tetrodonta and Eucalyptus miniata saplings in a north Australian savanna. *Plant Cell & Environment,* 23**,** 955-965.

ROBERTS, S., VERTESSY, R. & GRAYSON, R. 2001. Transpiration from Eucalyptus sieberi (L. Johnson) forests of different age. *Forest Ecology and Management,* 143**,** 153-161.

TAYLOR, D. & EAMUS, D. 2008. Coordinating leaf functional traits with branch hydraulic conductivity: resource substitution and implications for carbon gain. *Tree Physiology,* 28**,** 1169-1177.

TESKEY, R. O. & SHERIFF, D. W. 1996. Water use by Pinus radiata trees in a plantation. *Tree Physiology,* 16**,** 273-279.

VERTESSY, R. A., BENYON, R. G., O'SULLIVAN, S. K. & GRIBBEN, P. R. 1995. Relationships between stem diameter, sapwood area, leaf area and transpiration in a young mountain ash forest. *Tree Physiology,* 15**,** 559-567.

VERTESSY, R. A., HATTON, T. J., REECE, P., O'SULLIVAN, S. K. & BENYON, R. G. 1997. Estimating stand water use of large mountain ash trees and validation of the sap flow measurement technique. *Tree Physiology,* 17**,** 747-756.

VERTESSY, R. A., WATSON, F. G. R. & O′SULLIVAN, S. K. 2001. Factors determining relations between stand age and catchment water balance in mountain ash forests. *Forest Ecology and Management,* 143**,** 13-26.

WESTOBY, M. & WRIGHT, I. J. 2003. The leaf size - twig size spectrum and its relationship to other important spectra of variation among species. *Oecologia,* 135**,** 621-628.

WHITE, D., BEADLE, C., WORLEDGE, D., HONEYSETT, J. & CHERRY, M. 1998. The influence of drought on the relationship between leaf and conducting sapwood area in Eucalyptus globulus and Eucalyptus nitens&lt;/i&gt. *Trees - Structure and Function,* 12**,** 406-414.

YUNUSA, I. A. M., AUMANN, C. D., RAB, M. A., MERRICK, N., FISHER, P. D., EBERBACH, P. L. & EAMUS, D. 2010. Topographical and seasonal trends in transpiration by two co-occurring Eucalyptus species during two contrasting years in a low rainfall environment. *Agricultural and Forest Meteorology,* 150**,** 1234-1244.

ZEPPEL, M. & EAMUS, D. 2008. Coordination of leaf area, sapwood area and canopy conductance leads to species convergence of tree water use in a remnant evergreen woodland. *Australian Journal of Botany,* 56**,** 97-108.
